# Optimization of Influential Nutrients during Direct Cellulose Fermentation into Hydrogen by *Clostridium thermocellum*

**DOI:** 10.3390/ijms16023116

**Published:** 2015-01-30

**Authors:** Rumana Islam, Richard Sparling, Nazim Cicek, David B. Levin

**Affiliations:** 1 Department of Biosystems Engineering, University of Manitoba, Winnipeg, MB R3T 2N2, Canada; E-Mails: rislam1975@gmail.com (R.I.); nazim.cicek@umanitoba.ca (N.C.); 2 Department of Microbiology, University of Manitoba, Winnipeg, MB R3T 2N2, Canada; E-Mail: richard.sparling@umanitoba.ca

**Keywords:** cellulose, *Clostridum thermocellum*, medium composition, optimization, central composite design

## Abstract

Combinatorial effects of influential growth nutrients were investigated in order to enhance hydrogen (H_2_) production during direct conversion of cellulose by *Clostridium thermocellum* DSM 1237. A central composite face-centered design and response surface methodology (RSM) were applied to optimize concentrations of cellulose, yeast extract (YE), and magnesium chloride (Mg) in culture. The overall optimum composition generated by the desirability function resulted in 57.28 mmol H_2_/L-culture with 1.30 mol H_2_/mol glucose and 7.48 mmol/(g·cell·h) when cultures contained 25 g/L cellulose, 2 g/L YE, and 1.75 g/L Mg. Compared with the unaltered medium, the optimized medium produced approximately 3.2-fold more H_2_ within the same time-frame with 50% higher specific productivity, which are also better than previously reported values from similar studies. Nutrient composition that diverted carbon and electron flux away from H_2_ promoting ethanol production was also determined. This study represents the first investigation dealing with multifactor optimization with RSM for H_2_ production during direct cellulose fermentation.

## 1. Introduction

Rising global concerns about climate change coupled with accelerated energy consumption has propelled the search for clean and sustainable alternatives to fossil fuels. Biologically generated hydrogen derived from non-food residues originated from agro-industrial and forestry activities as feedstock holds great promise as recognized by the scientific community [1]. Direct microbial conversion of pre-treated cellulosic biomass offers an attractive option to avoid expensive enzymatic saccharification processes releasing soluble sugars [2,3,4,5]. Culture suspensions with high concentrations of solids allow enhanced process efficiency by reducing water separation cost, increasing product concentrations, and lowering energy inputs. Fermentation of lignocelluloses under high loading conditions was attempted via simultaneous saccharification and fermentation (SSF) processes [6,7]. Despite a ten-fold reduction in the cost of cellulase enzyme production, the projected cost for consolidated bioprocessing (CBP) was estimated to be lower than SSF and similar configurations requiring dedicated enzyme production [2,3,5]. However, technological challenges such as low volumetric production rates and low product-tolerance of fermenting organisms impose major bottlenecks to commercialize cellulosic H_2_ production through CBP and substantial research efforts are required for successful commercialization [3,5].

*Clostridium thermocellum* is a thermophilic, cellulolytic bacterium that has been extensively studied for its efficient depolymerization of crystalline cellulose into hydrogen, ethanol and organic acids as fermentation end-products [8,9,10,11]. Hydrogen production rates and yields are regulated by the carbon-flow distribution among various metabolic pathways, which are greatly influenced by the culture environment. Growth nutrient composition, a crucial component of the microbial environment, needs to be designed with care as it determines the economy and efficiency of a process to a great extent. To date, considerable research efforts have been directed towards optimization of biohydrogen production [12,13,14] from soluble feedstock, but none involved direct fermentation of cellulose.

Traditional optimization methods by varying one-factor-at-a-time are not only inefficient but also ineffective in their detection of possible interactions between factors that are often significant. Statistically designed experiments require minimum numbers of replicates to generate enough information about effects of main factors and possible interactions among them. A number of studies that applied statistical optimization to improve fermentative hydrogen or ethanol production using mixed and pure cultures [13,14,15,16] considered a wide variety of factors which fell into two categories: (i) medium nutrients such as carbon source, minerals, salts; and (ii) process parameters such as temperature, initial pH, and incubation-time. In some processes, optimization of one response may deteriorate the performance of other competitive responses. A multi-response optimization approach such as the method of desirability [17] is useful in this scenario. Based on specified ranges of multiple responses, the desirability function determines a set of conditions that produce adequately balanced optima for all responses involved.

Cellulose, yeast extract (YE), and magnesium were identified as most influential by a screening design involving seven different growth medium constituents of *C. thermocellum* DSM 1237 [18]. Magnesium ions (Mg) are the most abundant divalent cations in living cells acting as an essential co-factor for many enzymes involved in glycolysis [19]. A number of previous studies reported positive effects of elevated Mg [20,21] and YE concentrations [22,23] on H_2_ or ethanol production. It was reported that YEPD (yeast extract peptone dextrose) medium supplemented with 0.5 mM magnesium exhibited a 40% higher rate of fermentation than the control [20]. Ethanol fermentation under heat and ethanol stress by *Zymomonas mobilis* showed dramatic improvement after 10–20 mM magnesium was added [21]. However, excessively high concentrations (125 mM MgCl_2_) were found to be inhibitory to the growth of *C. thermocellum* JW20 [24].

In the current study, combinatorial effects of three medium components were explored and an optimum composition for H_2_ production was developed with the aid of response surface methodology (RSM), based on a central composite face centered (CCF) design. Individual and joint effects of these nutrients were investigated to allow deeper insights into growth nutrients requirement of *C. thermocellum* for one-step conversion of cellulose into H_2_. We considered YE as a standard ingredient representing complex growth nutrients that allows other non-standard sources such as distillery by-products, paper mill effluents, corn steep liquor *etc.* to be calibrated against YE based on their availability and cost. Other process optimization approaches such as fed-batch or semi-continuous mode of fermentation with recycling may substantially reduce the cost of YE addition by nutrient recovery.

## 2. Results and Discussion

Combinatorial effects of the three medium nutrients, cellulose, YE and Mg on volumetric and molar yields of hydrogen were studied with a CCF design. The primary response, concentration of H_2_, was presented in Table 1 along with substrate specific and cell-mass specific yields of H_2_ calculated based on total glucose equivalents converted into end-products (Gp). Concentration of ethanol, the major competing end-product, were also shown in this table. These experimental data collected based on the CCF design were applied to develop fitted polynomial models. An overall optimum medium composition was estimated based on these models that allows high concentration of H_2_ without severely compromising yields during direct fermentation of cellulose. Significant contributions of several two-factor interactions to estimated responses provided more insight into the direct cellulose conversion by *C. thermocellum*.

Other major soluble (acetate, lactate and formate) end-products of cellulose fermentation by *C. thermocellum* DSM 1237 are presented in Table 2 along with cell-biomass, redox (O/R) balance and substrate conversion (%) data. As shown by the cell-biomass data, growth was strongly stimulated by various nutrient combinations, which enhanced substrate conversion resulting in high concentration of end-products. Depending on nutrient combinations, the portion of initially added cellulose converted into end-products and cell-biomass varied widely (3.2% to 65.5%), meaning all cultures remained under excess-substrate conditions.

These experimental results represent only the first 20 h of fermentation without pH control, when the pH remained at or above 6.8. As our objective was to determine optimum medium compositions that enhance H_2_ production, we examined the impacts of various combinations of nutrients during the active phase of growth. We have applied these findings (ongoing work) in a pH controlled and open-to-atmosphere process where cellulose conversion was nearly complete (~98%).

**Table 1:** Design matrix of the central composite face-centered design with measured responses for H_2_ and ethanol. These data represent averages obtained from biological replicates.

Composition	Cellulose (A)	YE (B)	Mg (C)	H_2_			Ethanol (mmol/L)
	g/L			mmol/L	mol/(mol hexose)	mmol/(g·cell·h)	
1	10	1	1	17.83	1.22	5.09	10.8
2	50	1	1	12.71	1.19	4.05	8.8
3	10	2	1	18.90	1.17	5.49	12.71
4	50	2	1	15.34	1.14	3.91	10.86
5	10	1	2	31.84	1.04	6.46	33.21
6	50	1	2	23.40	0.98	4.46	24.92
7	10	2	2	46.22	1.11	6.67	50.4
8	50	2	2	43.40	1.02	5.03	45.17
9	10	1.5	1.5	41.79	1.22	6.59	35.6
10	50	1.5	1.5	42.50	1.17	5.86	39.74
11	30	1	1.5	33.77	1.29	6.34	26.9
12	30	2	1.5	50.77	1.27	5.68	39.8
13	30	1.5	1	22.50	1.15	4.26	17.1
14	30	1.5	2	43.80	1.05	5.25	45.3
Centre point	30	1.5	1.5	51.60	1.29	6.79	37.06
	30	1.5	1.5	47.47	1.25	6.61	40.82
	30	1.5	1.5	48.90	1.24	6.62	41.9

**Table 2:** Production of organic acids and cell-biomass by *C. thermocellum* DSM 1237 with corresponding redox balance and substrate converted into products.

Run No.	Acetate	Formate	Lactate	Cell Growth (mg·protein/L)	Redox (O/R)	Substrate Utilization (%)
	mmol/L					
1	10.65	1.89	3.86	177	1.11	23.82
2	6.98	2.33	1.96	163	1.16	3.20
3	11.94	3.16	3.79	181	1.14	23.65
4	9.1	3.93	2.36	210	1.19	4.17
5	18.3	3.36	3.91	270	1.10	50.85
6	13.84	3.36	2.56	289	1.12	7.69
7	22.22	5.35	1.96	338	1.19	65.52
8	24.1	5.68	4.95	492	1.17	13.71
9	21.04	3.19	3.98	355	1.14	55.76
10	22.65	1.97	1.47	409	1.21	10.59
11	15.37	3.04	2.80	294	1.18	12.05
12	25.43	2.00	3.34	510	1.27	22.25
13	11.16	3.44	4.30	291	1.20	8.61
14	24.34	6.44	3.39	475	1.24	23.07
Centre points	27.81	6.32	5.54	378	1.19	23.46
	23.49	5.18	2.62	405	1.23	21.67
	25.94	5.27	1.95	417	1.21	22.49

### 2.1. Modeling and Subsequent Optimization

For each response, quality of fit was compared among various model options such as, linear, quadratic or cubic based on their lack-of-fit *p*-values and *R*-squared values. The model with the best combination of *R*-squared, *F*-value, *p*-value, and lack-of-fit was considered for further analysis. The selected models were subjected to the stepwise process of model construction maintaining appropriate hierarchy of terms. ANOVA were applied along with the diagnosis of the fitted surface based on a set of criteria: (i) model significance (large *F*-value, *p*-value < 0.05); (ii) insignificant lack-of-fit (*p* > 0.01); (iii) adequate precision greater than 4; and (iv) behavior of residuals. The fitted models were then subjected to numerical optimization based on the simplex algorithm to obtain best possible combinations of predictor variables.

### 2.2. Concentration of H_2_

ANOVA indicated a good fit (*p* < 0.0001) of the reduced quadratic model selected with a high *F*-value of 44.53 and an insignificant *p*-value for the lack-of-fit (Table 3). This model reflected strong stimulatory influence of Mg and YE while high cellulose concentrations exerted a relatively weak negative effect. The interaction between cellulose concentration (A) and magnesium (C) was found insignificant and removal of the corresponding term (AC) improved the model while YE and Mg showed high interaction (BC). Significant contribution from second order terms indicates influence of quadratic effects of cellulose and magnesium. Negative coefficients of quadratic terms indicate the presence of a unique maximum zone close by.

**Figure 1:**
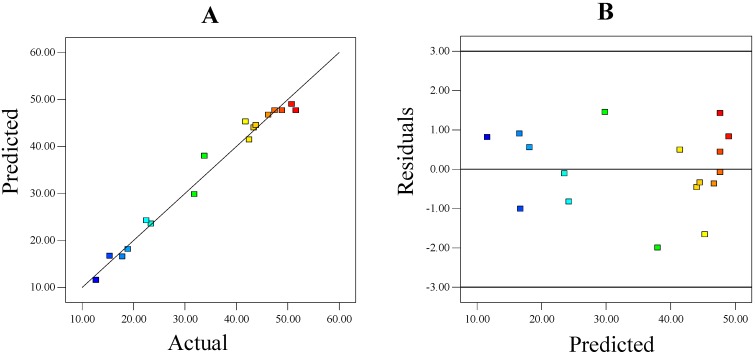
Diagnostic plots for the H_2_-concentration model. (**A**) Correlation between measured and predicted response; and (**B**) scatterings of residuals against predicted values of response.

Diagnostic plots for the H_2_-concentration model (Figure 1) resembled the quality of fit and adequacy of the regression model to navigate the design space. Here, the predicted *vs.* actual showed a good agreement between predicted and actual values of response. The residual *vs.* predicted plot is used to check for constant variation and zero mean of errors. The plot displayed no systematic trend of residuals and a fairly well-scattered pattern evenly distributed around the horizontal axis.

**Table 3:** Reduced quadratic model obtained for concentration of H_2_. Model co-efficients are presented in terms of coded factors.

Source	Co-Efficient	*F*-Value	*p*-Value
Model	–	44.53	<0.0001
Intercept	47.68	–	–
A: cellulose	−1.92	4.51	0.0665
B: YE	5.51	36.97	0.0003
C: Mg	10.14	125.25	<0.0001
AB	0.9	0.79	0.4014
BC	3.84	14.34	0.0053
A^2^	−4.3	6.05	0.0393
B^2^	−4.18	5.7	0.044
C^2^	−13.3	57.75	<0.0001
Lack of Fit	–	2.15	0.3506

*R*^2^ = 0.98; *R*^2^ (predicted) = 0.90; *R*^2^ (adjusted) = 0.96; adequate precision = 18.19.

### 2.3. Substrate Specific Yield and Specific Productivity of H_2_

A reduced quadratic model was found to show the best fit for substrate specific yields (mol H_2_/mol hexose) of H_2_ (Table 4). YE was detected to have a suppressing effect on hydrogen yields. Also interactions between cellulose and Mg and between YE and Mg were identified to be significant.

**Table 4:** Reduced quadratic model obtained for molar yields of H_2_. Model co-efficients are presented in terms of coded factors.

Source	Co-Efficient	*F*-Value	*p*-Value
Model	–	22.95	0.0002
Intercept	1.26	–	–
A: cellulose	−0.026	8.18	0.0244
B: YE	−0.0086	0.91	0.3715
C: Mg	−0.067	55.17	0.0001
AB	−0.0041	0.16	0.6981
AC	−0.011	1.27	0.2976
BC	0.028	7.48	0.0291
A^2^	−0.06	11.97	0.0106
B^2^	0.064	13.23	0.0083
C^2^	−0.15	76.11	<0.0001
Lack of Fit	–	1.07	0.5478

*R*^2^ = 0.96; Adj. *R*^2^ = 0.92; Pred. *R*^2^ = 0.81; adequate precision: 15.54.

A reduced quadratic model revealed that negative impact of high cellulose concentration was the most prominent main effect on the specific productivity of H_2_ while enhancing effect of magnesium was the second significant main effect to the model (Table 5). All two-factor interactions and two quadratic terms were found insignificant and removal of those terms improved the model fitness.

**Table 5:** Reduced quadratic model obtained for the specific productivity of H_2_. Model co-efficients are presented in terms of coded factors.

Source	Co-Efficient	*F*-Value	*p*-Value
Model	–	24.97	<0.0001
Intercept	6.36	–	–
A: cellulose	−0.72	36.38	<0.0001
B: YE	0.018	0.024	0.8807
C: Mg	0.49	16.68	0.0015
C^2^	−1.27	46.79	<0.0001
Lack of Fit	–	16.75	0.058

*R*^2^ = 0.89; Adj. *R*^2^ = 0.86; Pred. *R*^2^ = 0.80; adequate precision: 15.72.

### 2.4. A Balanced Optimum through Desirability

A trade-off exists between product yield, favored by low substrate concentration, and product concentration, favored by high substrate concentrations. To obtain a suitable combination of yields and rates, the desirability function was applied to H_2_ models for concentration, substrate-specific yield and specific productivity. To apply the overall desirability (D) function, “importance” values were assigned to maximize the volumetric H_2_ production while keeping both molar yields and specific productivities within their ranges (Table 6). The best overall solution generated by the D function (Section 3.6) predicted 52.82 mmol H_2_/L with 1.24 mol H_2_/mol glucose and 6.4 mmol/(g·cell·h) when predictor variables were at approximately 25 g/L cellulose, 2 g/L YE, and 1.75 g/L Mg. This composition is referred to as OptH (overall optimum for H_2_ production) from this point forward.

**Figure 2:**
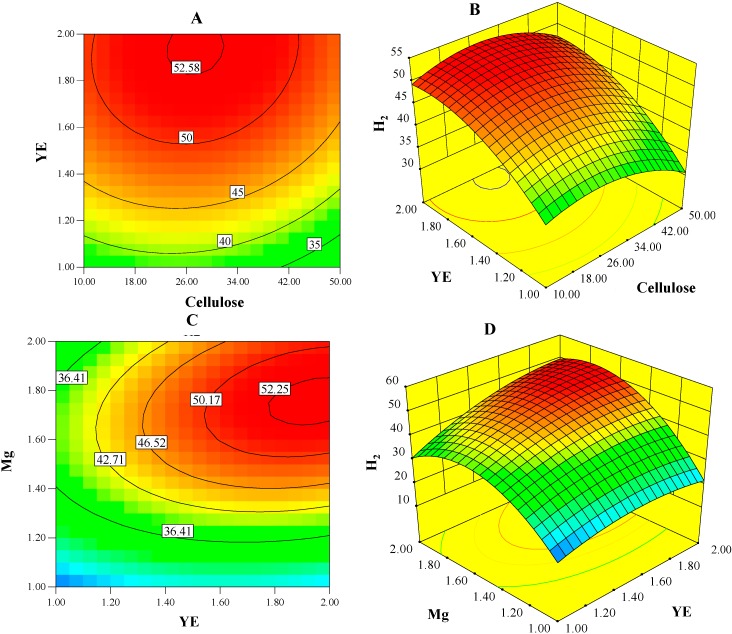
Contour and surface plots of H_2_-concentration (mmol/L) model show interactions between predictor variables. (**A**,**B**) show the interactions between yeast extract (YE) and cellulose while magnesium chloride (Mg) level was held constant, at 1.5 g/L; (**C**,**D**) show the interactions between Mg and YE while cellulose level was held constant, at 30 g/L.

Figure 2 displays interactions among predictor variables in surface and contour plots for concentration of H_2_ predicted by the D function. These plots show that the optimum point is located close to the boundary of the design region, in particular for the range of YE. With additional experiments we examined (one-factor-at a time method) whether elevated concentrations of YE allows significantly higher concentration of H_2_. In those experiments, the OptH composition was supplemented with up to 6 g/L YE and resulted in only about 15% more H_2_. Moreover, this additional YE will not only negatively impact the cost of medium, but also the residual YE (up to 60%) in the culture broth will create a high COD (chemical oxygen demand)-waste stream in the full-scale operation. Based on these observations, we conclude that the current range of YE is optimal.

**Table 6:** Components of the desirability function applied to obtain a balanced optimum for H_2_ production.

Predictor/Response Variable	Goal	Lower Limit	Upper Limit	Importance	Best Solution	Desirability
Cellulose (g/L)	Within range	10	50	3	25.33	0.95
Yeast extract (g/L)	Within range	1	2	3	1.94	0.95
Magnesium chloride (g/L)	Within range	1	2	3	1.75	0.95
Concentration (mmol H_2_/L culture)	Maximize	12.71	55	5	52.82	0.95
Substrate specific yield (mol H_2_/mol glucose)	Within range	0.98	1.31	3	1.24	0.95
Specific productivity (mmol H_2_/(g·protein·h)	Within range	3.91	6.79	3	6.4	0.95

### 2.5. Ethanol: The Major Competing End-Product

Besides H_2_, ethanol is another primary product of *C. thermocellum* during the exponential phase of growth on cellulose. Ethanol synthesis competed directly against H_2_ plus acetate production [9,18]. In the following sections we identified a medium composition that stimulates ethanol production directing carbon-flux away from H_2_ for this organism. Knowing the optimum composition for ethanol production would allow us to maintain nutrient concentration within ranges that minimize undesired products.

#### 2.5.1. Ethanol Concentration Model

For ethanol concentration, a quadratic model was applied for further analysis as it provided the best quality of fit (Table 7). ANOVA indicated high significance for this model with non-significant lack of fit and high *R*^2^ values. All main and quadratic effect terms showed high impact on ethanol concentration. Two out of three interaction terms were also detected to have significant contribution to the model. Model diagnostics resembled good agreement between actual and predicted responses and a random pattern of residuals (data not shown).

**Figure 3:**
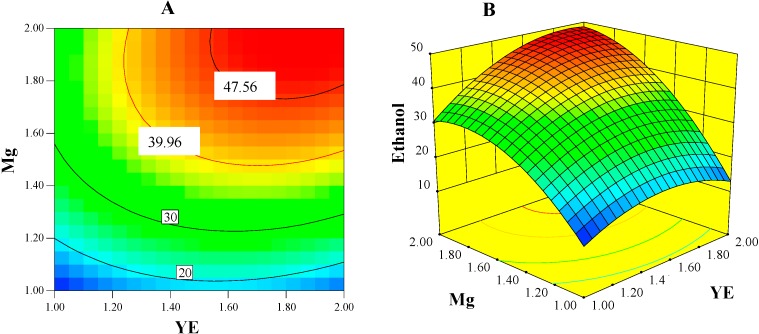
Contour (**A**) and surface (**B**) plots of ethanol-concentration (mmol/L) model show the interactions between YE and Mg when the level of cellulose was held constant at 30 g/L.

Figure 3A,B presents contour and surface plots for the ethanol concentration model. These plots at a constant cellulose concentration (30 g/L) showed strong joint effects of Mg–YE (BC) that enhanced ethanol concentration. The concentration model was subjected to numerical optimization procedure to obtain factor-combinations that allows for the maximum ethanol concentration. The optimum combination of 22.41 g/L cellulose, 2 g/L YE, and 2 g/L Mg was predicted by the ethanol concentration model to result in 48.95 mM ethanol. This combination is referred as OptE (optimum composition for ethanol production) combination from this point forward.

**Table 7:** Reduced quadratic model obtained for ethanol concentration. Model co-efficients are presented in terms of coded factors.

Source	Co-Efficient	*F*-Value	*p*-Value
Model	–	73.19	<0.0001
Intercept	39.16	–	–
A: cellulose	−1.32	2.94	0.1203
B: YE	5.43	49.61	<0.0001
C: Mg	13.87	323.71	<0.0001
AC	−1.21	1.97	0.1944
BC	4.18	23.55	0.0009
B^2^	−6.16	19.32	0.0017
C^2^	−8.31	35.17	0.0002
Lack of Fit	–	0.9	0.620

*R*^2^ = 0.99; *R*^2^ (predicted) = 0.97; adequate precision = 24.66.

#### 2.5.2. Stationary Point Estimation

Both the predicted optimum combination and response surface plots in Figure 3 indicated that the point for predicted optimum ethanol concentration is located on the boundary of the design region. Based on this observation, it can be assumed that the true optimum was missed and higher levels of YE and Mg would allow improvement in ethanol concentration. Since application of the steepest ascent method is not appropriate for a second order model, the stationary point was estimated to verify this assumption. Stationary point of this response surface is a local maxima where ethanol concentration stops improving with respect to all three predictor variables [17]. This stationary point, in terms of original values of three variables was found to be 21 g/L cellulose, 2.13 g/L YE and 2.27 g/L Mg and the estimated response at this point was predicted to be 52.58 mmol/L ethanol. However, this ethanol concentration is not significantly different from that obtained from the OptE composition determined earlier. Moreover, supplementation of Mg was tested by adding up to 6 g/L of MgCl_2_·6H_2_O to the OptE composition (Figure S1, supplementary materials). No significant improvement in ethanol concentration over OptE was observed.

### 2.6. The Acetyl-CoA Branch Point

Based on the biochemical pathway of cellodextrin metabolism by *C. thermocellum*, pathways for both H_2_ (with acetate) and ethanol production descend from the same acetyl-coA branch-point [25]. This implies that these two reduced end-products compete for electrons arriving at this juncture. Nevertheless, a rise in H_2_ partial pressure (>50 kPa) directs metabolism away from acetate plus hydrogen toward other reduced end-products such as ethanol [26]. Metabolic shifts also occur due to changes in operational parameters also, such as stirred *vs.* unstirred conditions [27] or in co-cultures [22,28] through interspecies H_2_ transfer. We intended to formulate nutrient compositions that promote concentration and/or yields of H_2_. Besides, we also identified compositions that drive carbon and electron away from H_2_ favoring ethanol production. OptH and OptE combinations identified through optimization were not highly contrasting, since concentration of Mg is the main difference found between these two compositions. Both of these compositions aimed to maximize concentration of either H_2_ or ethanol were governed by a common factor during the exponential phase, cell-growth. This indicated that a general growth-rate enhancement (Table 3) was primarily responsible for higher production rates of both H_2_ and ethanol.

Moreover, as a consequence of growth enhancement, only the concentration of H_2_ increased while yields decreased in the presence of high magnesium. This implies that under high magnesium condition, the flow of carbon toward acetyl-coA was increased and comparative distribution from this branch-point favored ethanol over the acetate-H_2_ pathway. In addition, cell specific productivities of both H_2_ and ethanol were enhanced by increased level of magnesium. YE, a source of complex nitrogen (amino acids and nucleotides) and many micronutrients, supported higher molar yield of H_2_ only under low magnesium condition (Table 1).

### 2.7. Influence of Nutrients

In a preliminary investigation [18], seven nutrients were varied simultaneously to identify a subset of the most influential nutrients where Mg showed negative impacts at a high level. However, in the current study, a higher order design allowed unbiased estimation of factor-effects revealing positive influence of Mg. Also, keeping other divalent cations (Fe^2+^ and Ca^2+^) fixed at their low levels might have played a role in reversing the effect of Mg. The stimulatory effect of Mg observed here is not surprising as it is an essential macronutrient for carbohydrate metabolism and synthesis of proteins, lipids and nucleic acids. In the present study, magnesium ion concentration examined by the CCF design ranged between 4.9 and 9.8 mM and inhibitory effects to growth and production of *C. thermocellum* DSM 1237 were observed after Mg levels in cultures exceeded 25.6 mM concentration (Figure S1 in supplementary materials).

The interactive effects of Mg–YE showed a large positive influence on ethanol concentration in *C. thermocellum* culture as depicted by the corresponding model term (Table 7). This is possibly due to an interdependent growth stimulation effect induced by these two nutrients, which can be inferred from the cell biomass data (Table 2). The only report on multifactor optimization of biofuels production from direct cellulose fermentation considered five medium nutrients [15] filter paper (10–50 g/L), corn steep liquor (2–10 g/L), cysteine HCl (0.1–0.5 g/L), FeSO_4_·7H_2_O (0.01–0.05 g/L) and MgCl_2_·6H_2_O (0.5–2.5 g/L) for ethanol production by *C. thermocellum* SS19. They concluded that two main factors, cysteine and magnesium, including all of the 2-factor interactions, are insignificant. This implies level setting of each factor considered in design is the crucial part since main and interactive effects are highly dependent on ranges chosen for each factor. Also, one nutrient set at an excessively high or low level can diminish or amplify the effect of others. For example, beside a small amount of magnesium, addition of YE also introduces other metal ions such as iron, copper, and zinc [29]. With varied concentrations of YE, availability of these cations also varies in the medium and may collectively influence enzyme activities and/or growth of cells. In this case, additional experimentation with appropriate controls will be required to identify their effects.

### 2.8. Verification of Overall Optimum for H_2_ Production (OptH)

The predicted overall optimum composition for H_2_ production *i.e.*, the OptH, was verified using biological triplicates. Experimental outcome was in good agreement with the model prediction (Table 8). Relative to the unaltered condition (composition #1 in Table 1), concentration and productivity of H_2_ were about 3.2-fold and 1.5-fold higher respectively for the OptH, however, yields (mol H_2_/mol glucose) remained unchanged. This is not unexpected since a common characteristic of fermentative H_2_ production is that there is an inverse relationship between volumetric production rates and molar yield, and that volumetric production of H_2_ increases as the substrate concentration increases, but usually accompanied by a decrease in molar yield. This phenomenon was demonstrated during hydrogen production from both soluble and insoluble carbon substrates [9,10]. Also, irrespective of culturing mode (batch or continuous), increased organic loading rate was associated with decreased yields [30,31]. Relative to a previously reported yield of 1.28 mol H_2_/mol glucose-equivalent with 5 g/L of cellulose [9], the identical strain resulted in similar molar yields with about 5-fold higher initial cellulose concentration in OptH. In this respect, yields produced by the OptH are well within expectation. At this high cellulose concentration, the maximum specific productivity of H_2_ achieved in this study was higher compared with those reported to date [32] for monocultures of cellulose degrading species.

**Table 8:** Verification of the overall optimum composition (OptH) for H_2_ production.

Predicted *vs.* Actual	Concentration (mmol/L)	Specific rate (mmol/(g·cell·h))	Yield (mol/mol hexose)
Prediction	52.82	6.4	1.24
Experimental	57.28 ± 4.8	7.48 ± 1.2	1.30 ± 0.05

## 3. Experimental Section

### 3.1. Microorganism and Growth Medium

*Clostridium thermocellum* DSM 1237 (synonymous collection numbers include ATCC 27405, JCM 12338, and NCIB 10682) obtained from the German Collection of Microorganisms and Cell Cultures (DSMZ) was used throughout this study. After two subcultures, aliquots of the DSMZ culture were stored in glycerol at −80 °C and were revived before experimentations. For the growth and maintenance of the *C. thermocellum* culture, a modified version of the defined MJ nutrient medium [33] was prepared by adding yeast extract (YE) to the basal medium (per liter: 1.5 g KH_2_PO_4_, 2.9 g K_2_HPO_4_, 10.0 g MOPS, 150 mg CaCl_2_·2H_2_O, 1 g MgCl_2_·6H_2_O (g), 1.25 mg FeSO_4_·6H_2_O, 2.1 g urea, 1.0 mg resazurin and 3.0 g sodium citrate, 1 g YE, 20 mg biotin, 200 mg pyridoxamine–HCl, 40 mg *p*-aminobenzoic acid and 20 mg cyanocobalamin). This medium composition is referred to as the basic or unaltered medium in the Discussion section.

To prepare experimental vials for various combinations, cellulose was first dispensed to each vial at desired concentrations followed by addition of YE or Mg from concentrated (50×) stocks. Then a constant volume of 2× mineral solution, prepared with the remaining components of the medium, was dispensed using water as the make-up volume. Cysteine–hydrochloric acid was added to 1 g/L final concentration after making bottles anaerobic by repeated gassing with nitrogen and degassing cycles as described previously [9]. A 100-fold concentrated urea solution and vitamin solution were prepared separately and filter-sterilized into pre-sterilized and anaerobic bottles. Concentrated stock (100×) of filter sterilized cysteine-hydrochloric acid was added to each bottle. These solutions were added aseptically to each experimental bottle to the desired final concentrations before inoculation.

### 3.2. Experimental Set-up

For all experiments, serum bottles (60 mL) with 20 mL working volume were used in batch anaerobic fermentation at 60 °C. Starting pH values ranged from 7.35 to 7.42 and a shaking rate of 100 rpm was maintained in a water bath (Thermo Scientific Shaking Water Bath-SWB25, Thermo Fisher Scientific Inc., Waltham, MA, USA). The working-volume and shaking rate were selected based on trials preformed with 10 to 40 mL of work-volume and 50 to 200 rpm so that cellulose particles did not settle at the bottom or splash out from the medium during the agitation. From an exponential phase culture grown on the unaltered composition, inocula (10%) were added to each experimental unit and incubated at 60 °C for 20 h.

### 3.3. Experimental Design and Optimization

Three components of the growth medium of *C. thermocellum* were examined with the aid of a central composite face-centered (CCF) design and the overall optimum for hydrogen production was determined with the desirability function [17]. These components and their levels were selected based on screening experiments previously conducted with seven medium components [18].

### 3.4. Factor Coding

Three levels of coded factors represented the high (1), the low (−1), and the center-point (0) as specified in the experimental design (Table 9).

**Table 9:** Actual values and coded levels of factors used in the central composite face-centered (CCF) design.

Factors	Unit	Actual Values		
Coded Levels		Low (−1)	Centre (0)	High (1)
A: α-Cellulose	g/L	10	30	50
B: YE	g/L	1	1.5	2
C: Mg	g/L	1	1.5	2

Coding of factors were obtained using Equation (1):

(1) $$X_{i}=(x_{i}-x_{i0})/\Delta x_{i};\;i=1,\;2,\;3$$

where, *X_i_* is the dimensionless value of the *i*-th independent variable, *x_i_* is the corresponding natural value of that variable, *x*_*i*0_ is the natural value of that variable at the center of the design region and ∆*x_i_* is the increment of *x_i_*, *i.e.*, 1 unit of *X_i_*.

### 3.5. Statistical Modeling

To define the relationship among the responses of interest and the independent or explanatory variables, a polynomial model with interactions and quadratic terms was applied, as expressed in Equation (2):

(2) $$Y=\beta_{0}+\sum_{i=1}^{k}\beta_{i}x_{i}+\sum_{i=1}^{k}\beta_{ii}x_{i}^{2}+\sum_{i<j}^{k}\sum_{j}\beta_{ij}x_{i}x_{j}+\varepsilon$$

where, *Y* is the response measured and *β* represents regression coefficients of main effects, squared effects, interactions between main effects, and *ε* is the random error term. Based on relative significance (*p*-values) estimated for each model terms, stepwise elimination was performed to improve the model-*R*^2^ until no further improvement could be achieved. Models obtained for each response were optimized for maximum values applying the optimization toolbox of Design-Expert software version 8.0.1 (Stat-Ease Inc., Minneapolis, MN, USA) and corresponding values of predictor variables were taken as optimum conditions. All design points were executed as independent biological triplicates and the mean values of responses were considered for analysis of variance (ANOVA).

### 3.6. Desirability Function

The desirability function is applicable to combine multiple responses into a single response to obtain the best possible trade-off. This function was used here to compute a balanced nutrient composition that allowed maximizing the concentration of H_2_ without severely compromising the substrate-specific yields and specific productivity. In this method, each estimated response (*y_i_*) is transformed to a desirability function (*d_i_*) as shown in Equation (3), where 0 ≤ *d_i_* ≤ 1:

(3) $$\begin{array}{l} \;d_{i}=0,\;ify_{i}\leq y_{\text{min}}\\ or\;d_{i}=\{{\left(\frac{y_{i}-y_{\text{min}}}{y_{\text{max}}-y_{\text{min}}}\right)}^{w},\;if\;y_{\text{min}}<y_{i}<y_{\text{max}}\\ or\;d_{i}=1,\;if\;y_{i}\geq y_{\text{max}} \end{array}$$

where, *y*_min_ is the minimum acceptable value of the response *y_i_*, *y*_max_ is the maximum value and *w* is a weight factor. Through the desirability approach, individual desirability of multiple responses is combined using the geometric mean (Equation (4)) to formulate the overall desirability (D):

(4) $$D={\left(d_{1}\times d_{2}\times \ldots d_{k}\right)}^{1/k}$$

where *k* denotes the number of responses being optimized. The Design-Expert software was used to calculate D that follows the numerical optimization routine and the closer the overall desirability value to unity the better all goals are met. Often, the maximum value of a response is stretched beyond the highest response observed, meaning that the optimum response produced by this function was generally better than the best response observed from experimental runs.

### 3.7. Analysis of End-Products and Growth

Product gas (H_2_ and CO_2_) concentrations were measured using a gas chromatograph (Model 8610C, SRI Instruments, Torrance, CA, USA) with corrections for dissolved product gases as detailed in Islam *et al.* [8]. Ethanol and organic acids (lactate, acetate, and formate) were quantified with the same system using an Aminex HPX 87H (300 × 7.8 mm) ion exclusion column (Bio-Rad, Hercules, CA, USA) fitted with Cation-H, micro-guard cartridge (40 × 4.6 mm) and a refractive index detector installed in an ion-chromatography system (Dionex ICS-3000, Sunnyvale, CA, USA). Ethanol was Mobile phase of the liquid chromatography was 0.004 N H_2_SO_4_ and the flow rate was maintained at 0.75 mL/min.

As an indirect measurement of growth, cellular protein was measured with Coomassie blue reagent by the method of Bradford after processing and extraction as described previously [8]. Absorbance of protein samples loaded in 96-well plates was measured at 595 nm wavelengths using a microplate spectrophotometer (Biotek Powerwave microplate reader). Cell dry weight was estimated based on cellular protein content measured from each sample [9].

### 3.8. Estimation of Product Yields and Redox Balance

In our experiments, we incubated cultures for only 20 h where significant amount of substrate remained unused, including some unquantifiable soluble sugars generated in the course of fermentation. Therefore, to exclude all unfermented sugars, substrate specific yields (mol/mol hexose) were obtained by dividing total moles of H_2_ or ethanol by the total glucose-equivalents (G_p_) converted into final end-products of fermentation and cell mass. Total moles of G_p_ was calculated with the aid of following relationship:

Moles of G_p_ = moles of (lactate + acetate + ethanol + cellmass)/2 (5)

Specific productivities (mmol/(g·protein·h)) were estimated based on cumulative moles of H_2_ or ethanol generated per hour for each combination with respect to cell-protein measured. For oxidation/reduction (O/R) balance of fermentation, first reduction number of each compound was calculated according to the method described by Johnson *et al.* (1931) [34]. Then the molar amount of each compound was multiplied with its reduction number. Finally, the total amount of oxidized compounds was divided by the total amount of reduced compounds to obtain O/R values.

## 4. Conclusions

Growth medium composition was effectively optimized using a response surface methodology, which significantly improved direct conversion of cellulose by *C. thermocellum* DSM 1237 into hydrogen under excess carbon conditions. Results obtained from experimental runs were very similar to those predicted by the overall desirability function. Compared with the basic medium composition, significant improvement in concentration and productivity were achieved through optimization while molar yields remained close to that from the unaltered condition. This study reports the first investigation involving medium optimization with RSM for hydrogen production during direct bioconversion of cellulose.

## Supplementary Materials

Supplementary materials can be found at http://www.mdpi.com/1422-0067/16/02/3116/s1.
